# Herpes simplex virus interferes with amyloid precursor protein processing

**DOI:** 10.1186/1471-2180-5-48

**Published:** 2005-08-18

**Authors:** Suzanne J Shipley, Edward T Parkin, Ruth F Itzhaki, Curtis B Dobson

**Affiliations:** 1 Faculty of Life Sciences, Moffat Building, University of Manchester, Manchester, M60 1QD, UK; 2 School of Biochemistry and Molecular Biology, University of Leeds, Leeds, W. Yorks, LS2 9JT, UK

## Abstract

**Background:**

The early events underlying Alzheimer's disease (AD) remain uncertain, although environmental factors may be involved. Work in this laboratory has shown that the combination of herpes simplex virus type 1 (HSV1) in brain and carriage of the APOE-ε4 allele of the APOE gene strongly increases the risk of developing AD. The development of AD is thought to involve abnormal aggregation or deposition of a 39–43 amino acid protein – β amyloid (Aβ) – within the brain. This is cleaved from the much larger transmembranal protein 'amyloid precursor protein' (APP). Any agent able to interfere directly with Aβ or APP metabolism may therefore have the capacity to contribute towards AD. One recent report showed that certain HSV1 glycoprotein peptides may aggregate like Aβ; a second study described a role for APP in transport of virus in squid axons. However to date the effects of acute herpesvirus infection on metabolism of APP in human neuronal-type cells have not been investigated. In order to find if HSV1 directly affects APP and its degradation, we have examined this protein from human neuroblastoma cells (normal and transfected with APP 695) infected with the virus, using Western blotting.

**Results:**

We have found that acute HSV1 (and also HSV2) infection rapidly reduces full length APP levels – as might be expected – yet surprisingly markedly *increases *levels of a novel C-terminal fragment of APP of about 55 kDa. This band was not increased in cells treated with the protein synthesis inhibitor cycloheximide

**Conclusion:**

Herpes virus infection leads to rapid loss of full length APP from cells, yet also causes increased levels of a novel 55 kDa C-terminal APP fragment. These data suggest that infection can directly alter the processing of a transmembranal protein intimately linked to the aetiology of AD.

## Background

The key events which initiate Alzheimer's disease (AD) remain unclear, though environmental factors have been shown to be involved [1]. The two main neuropathological features of AD – senile plaques (SP) and neurofibrillary tangles – occur also in the normal elderly. AD may be triggered when the numbers of these features increase to abnormal levels. Although there is much evidence supporting the involvement of one or both of these structures in the disease process, it is unclear whether they are involved directly, what processes underlie their formation, and why their numbers rise during the development of AD. However, any environmental stimulus capable of leading to production of the abnormal proteins which make up these structures might thereby contribute to the occurrence of AD.

β-amyloid protein (Aβ) is the major proteinaceous component of senile plaques, and the abnormal deposition of aggregates of this protein is thought to give rise to SP formation. Aβ comprises a sequence of 39–43 amino acids and is formed by cleavage from the much longer amyloid precursor protein (APP), first by β-secretase then by γ-secretase. Generation of Aβ may lead eventually to the development of neurotoxic SPs, which themselves cause further tissue damage and SP generation, or alternatively to the production of small neurotoxic Aβ assemblies, which may be the most damaging form of Aβ [2]. Whatever the mechanism leading to neuronal damage after Aβ generation, any agent which interferes with amyloid systems may thereby contribute to the development of AD.

Studies of the possible role of pathogens in AD were made possible in the early 1990s by the development of PCR, allowing detection of low levels of viral or bacterial DNA within human brain tissues. However very few investigators have undertaken such studies, and most work has focused on herpes simplex virus type 1 (HSV1). A causal role for this virus in triggering AD was suggested by Itzhaki et al., who found that the risk of developing AD associated with carriage of an APOE-ε4 allele depends on the presence of latent HSV1 in the brain [3,4]. Coupled with the finding here of a higher APOE-ε4 allele frequency amongst individuals who suffer damage after HSV1 reactivation in the periphery – seen as cold sores – this indirectly supports a role for HSV1, perhaps acting with other pathogens [5], as a key environmental contributor to AD [3,4].

Recent epidemiological studies support the involvement of a pathogen in AD: cognitive function in AD patients declines for at least 2 months after a systemic infection [6]; cognitive decline in elderly cardiovascular patients correlates with viral burden [7]. These could be explained by systemic infection causing brain inflammation which, in turn, leads to reactivation of latent HSV1 – and consequent further damage in the CNS. Consistently, in middle-aged controls, very few of whom would harbour HSV1 in brain [8], no cognitive decline occurs after systemic infection [9]. Enhancement of damage in brain, due to the presence of an infectious agent, is supported by the finding that inflammation in brain caused by lipopolysaccharide is augmented in pre-clinical prion-infected mice [10].

One mechanism by which HSV1 might contribute to AD was suggested by the detection of a sequence homology between Aβ and the HSV1 glycoprotein B (gB), a viral coat protein which is involved in attachment of the virus to cells, and by the finding that synthetic peptides derived from gB can give rise to Aβ-like aggregates. The authors suggested that such viral proteins might act as a seed for amyloid plaque formation [11]. Another way in which infection might affect amyloid biochemistry is suggested by studies demonstrating that Aβ or APP are upregulated in response to a wide range of injurious stimuli, including head injury [12], stroke [13] or HIV infection [14]. Increased expression of APP – though not of the related protein amyloid precursor-like protein 2 (APLP2) – has been reported in cutaneous wound repair [15]. APP foci colocalise with sites of opportunistic infection in HIV dementia patients, including sites of herpes virus (cytomegalovirus) infection [16]. Another type of APP-HSV1 interaction has been demonstrated in axonal transport: APP was found to be present in HSV1 particles and it was suggested that this could lead to alterations in location and processing of APP at the nerve terminal [17] causing synaptic and neuronal dysfunction.

No studies have been made on HSV1 effects on APP or Aβ in cells in culture. Here we describe the first to investigate whether levels of APP and its metabolites are affected by HSV1 infection, using human neuronal-type cells in culture.

## Results and discussion

**Figure 1:**
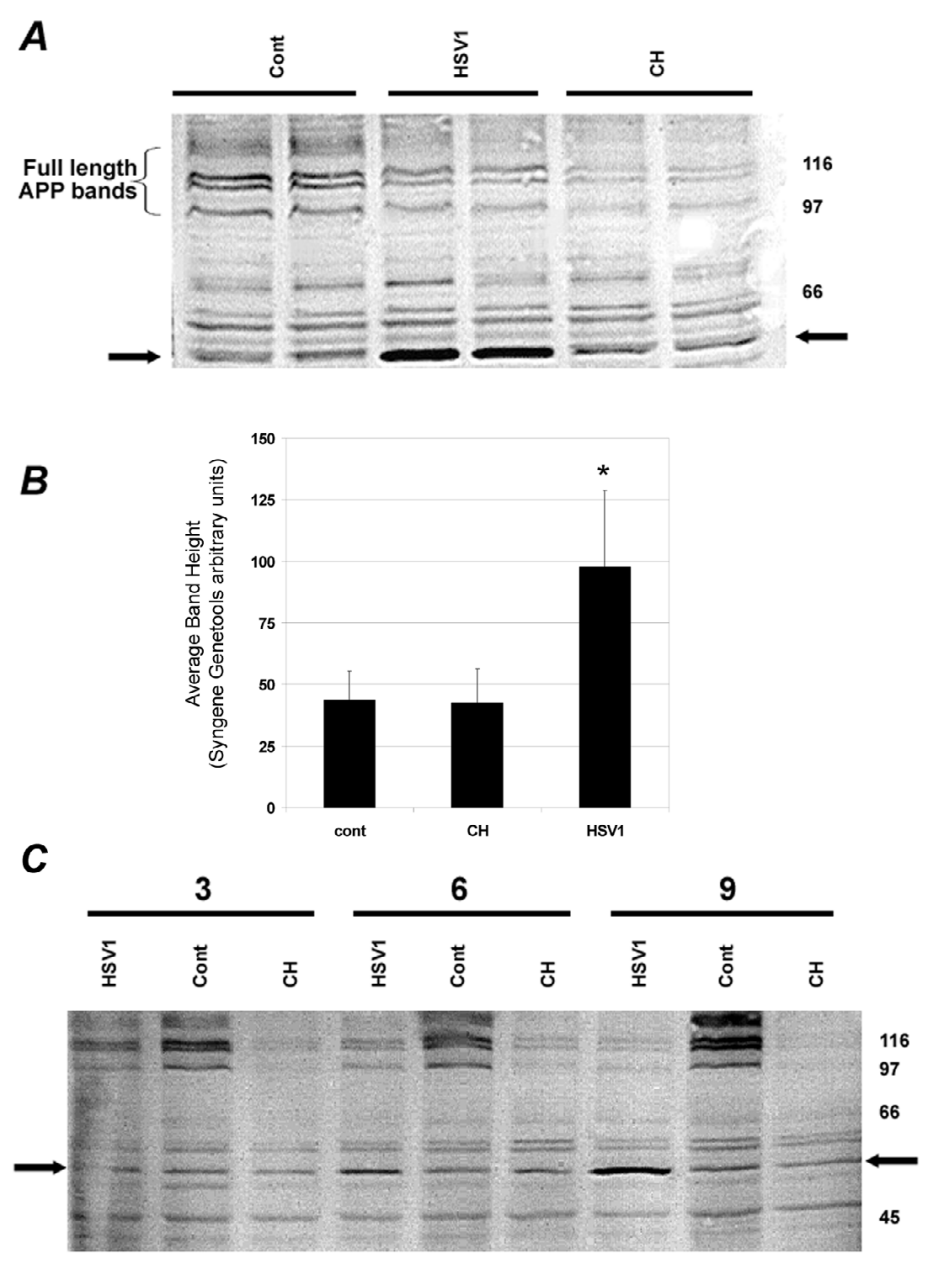
**Effect of HSV1 infection on APP processing in SHSY5Y cells**. (A) SHSY5Y human neuroblastoma cells were either untreated (cont), infected with herpes simplex virus type 1 (HSV1), or treated with the protein synthesis inhibitor cycloheximide (CH), then incubated for a further 6 h. Cell lysates were subjected to Western blot analysis, using an anti-C-terminal APP antibody (Sigma Aldrich A8717) at 1:4000 dilution. Each lane contains cell lysate prepared from a single flask (two flasks were used per treatment). Several full-length APP bands are clearly visible. Arrows indicate the band intensified by HSV1 infection. (B) Quantification of 55 kDa band in control (cont), cycloheximide-treated (CH), or HSV1 infected cells, assessed using Syngene GeneTools software. Values show average band height from five independent experiments for cells treated for 6 h, each involving two or four separately processed flasks. Bars show standard deviation. Treatments resulting in values significantly different from control are marked * (indicates p < 0.002; ANOVA). (C) Effect of time period after inoculation with virus on height of 55 kDa band. SHSY5Y human neuroblastoma cells were either untreated (cont), infected with HSV1, or treated with cycloheximide (CH), then incubated for a further 3, 6 or 9 h. Cell lysates were analysed by Western blotting as in Fig 1A. Arrows indicate the band intensified by HSV1 infection.

We carried out initial experiments on SHSY5Y cells to determine the level of HSV1 needed to ensure that on inoculation most cells were infected. We then examined levels of APP in lysates prepared from the cells 6 h after infection with this dose of HSV1. Western blots stained with anti C-terminal APP antibody (Fig. 1A) revealed, as expected, various full-length APP bands in uninfected cells corresponding to the three main isoforms in multiple glycosylation states. Deglycosylation experiments have shown that the lower single band (100 kDa) is immature APP695, that the doublet above this (approx molecular weight 115 kDa) is mature APP695 and immature APP751/APP770 and that the highest molecular weight group (approx. 125 to 135 kDa) comprises a mixture of mature APP751 and mature APP770 (Parkin et al., unpublished observations).

The intensity of these bands decreased appreciably in cells harvested only 3 h after inoculation with HSV1, as did those from cells treated with the protein synthesis inhibitor cycloheximide. This decline after infection may reflect loss of ability of the cells to generate new APP due simply to virus-induced shut-down of protein synthesis [18].

Of particular interest was the relative intensity of a 55 kDa band, again apparent after C-terminal APP antibody staining. This band was similarly intense in control-treated and cycloheximide-treated cells, but had far greater intensity after HSV1 infection. This increase in HSV1-infected cells alone was highly reproducible, occurring in many experiments, each carried out independently, from the infection to the blotting stage. Band intensity for cells treated for 6 hours was quantified using Syngene Genetools software and averaged over five such experiments is shown in Figure 1B; value for HSV1-infected cells was significantly different from control, being elevated by 124% (95%CI 101 – 148%; p < 0.002), whereas that for cycloheximide-treated cells was similar to the control value.

To exclude the possibility that the band reflected synthesis of a viral protein which cross-reacts non-specifically with the antibody, we immunostained blots produced from gels of viral proteins from our HSV1 preparations. No evidence for any cross-reaction of the Sigma C-terminal APP antibody with these proteins could be found, suggesting that the strengthened band was cell-derived (results not shown). This result does not preclude any cross-reactivity with an ICP, as the latter would not be present within virions, but the presence of the 55 kDa protein in uninfected cells, and also, at a higher level, in APP-transfected cells (see below), strongly supports a cellular, non-viral origin of the protein. Interestingly, APP has been detected in HSV1 virions by Western blotting [17], but this probably reflects the authors' usage of a much more concentrated and purified virus preparation than that of the inoculate we use for infecting cells.

We next examined the influence of duration of infection on these viral-induced changes (Fig. 1C). As anticipated, with increasing duration before harvesting, the depletion of APP became more pronounced (this was found also with cycloheximide treatment); in contrast, the intensity of the C-terminal 55 kDa fragment seen in the infected cells was not higher than in uninfected cells at 3 hr, but increased at 6 hr and 9 hr. However there was minimal further change as incubation increased to 24 hr (not shown). This result also was found to be reproducible on repeating the experiment several times from infection to blotting. It suggests that infection prevents synthesis of APP, leading to its gradual depletion, but causes also the abnormal accumulation of the 55 kDa fragment due, presumably, to a decrease in degradation of the latter. However, for longer incubation times, we cannot exclude the possibility of sequestration of some APP within newly synthesised viral particles, as reported elsewhere [17], which might reduce the amount of APP in the virus-free cell lysates used for the preparation of cell proteins.

**Figure 2:**
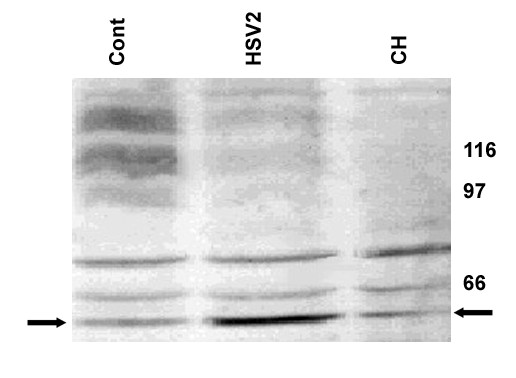
**Effect of HSV2 infection on APP processing in SHSY5Y cells**. SHSY5Y human neuroblastoma cells were either untreated (cont), infected with HSV2, or treated with cycloheximide (CH), then incubated for 9 h. Cell lysates were analysed by Western blotting as in Fig 1A. Arrows indicate the band intensified by HSV2 infection.

We repeated these experiments with herpes simplex virus type 2 (HSV2), to see whether these effects were specific to HSV1. Fig. 2 shows that very similar findings were obtained, with 55 kDa band intensity for HSV2-infected cells 9 hr after infection being 101% greater than that for control cells (95%CI 94 – 107%; p < 0.005) suggesting that this phenomenon is not uniquely associated with HSV1 infection.

**Figure 3:**
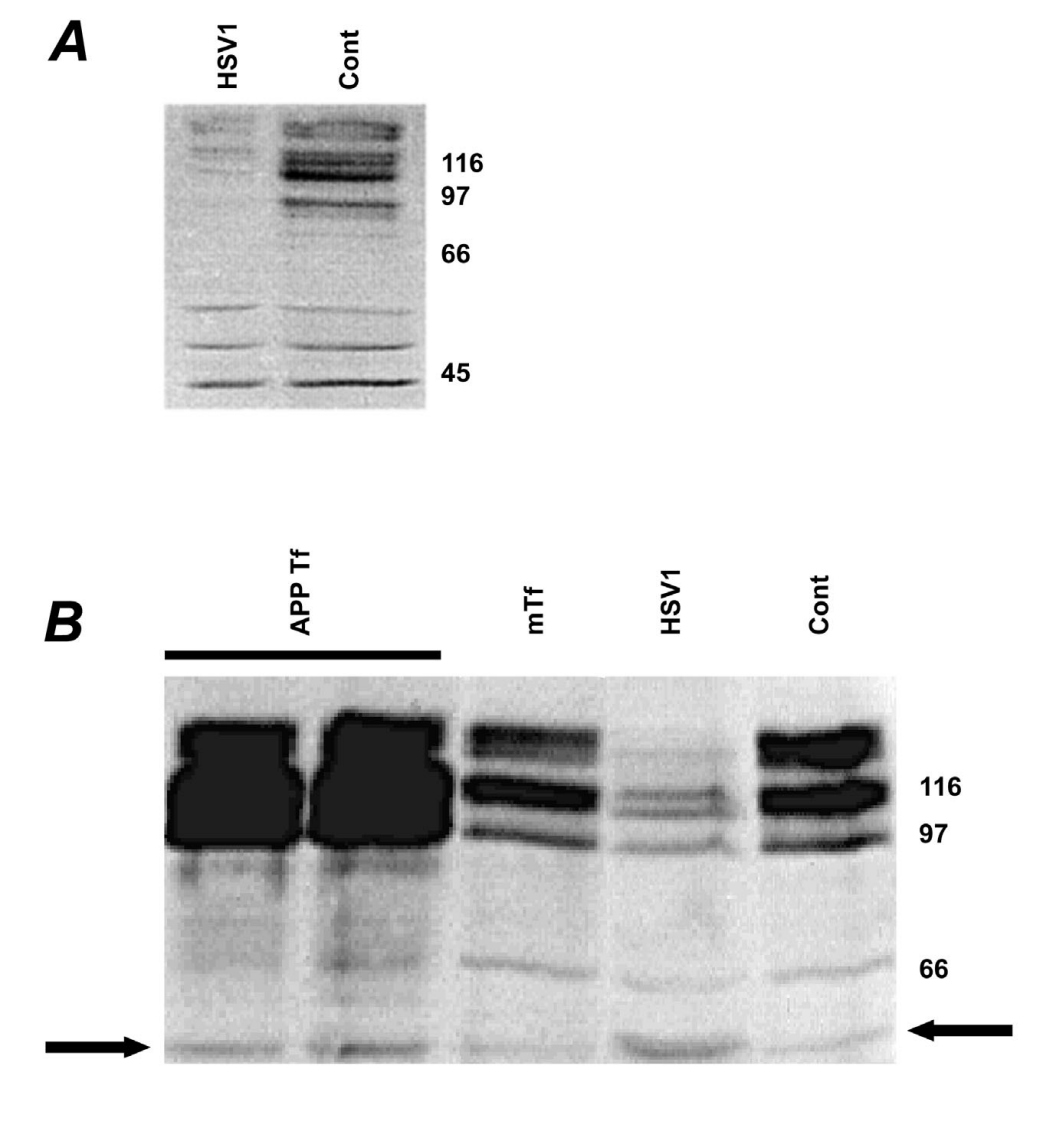
**Absence of N-terminal fragment and confirmation that 55 kDa fragment is derived from APP**. (A) Staining with antibody to N-terminal APP. SHSY5Y human neuroblastoma cells were either untreated (cont) or infected with HSV1, then incubated for a further 6 h. Cell lysates were analysed by Western blotting using an anti-N-terminal APP antibody 22C11. No evidence for increase in an N-terminal fragment of the expected M Wt. was observed, suggesting the latter may be metabolised further or secreted. (B) Strengthening of 55 kDa band in uninfected APP-transfected SHSY5Y cells. Normal SHSY5Y human neuroblastoma cells, mock-transfected SHSY5Y cells (mTf) or APP695 transfected cells (APP Tf) were uninfected prior to harvest. In addition normal SHSY5Y cells which had been infected with HSV1 (lane labelled HSV1) for 6 h were also prepared, to allow position of the HSV1-senstive 55 kDa band to be clearly identified. Cell lysates were subjected to Western blot analysis, and probed with an anti-C-terminal APP antibody (Sigma Aldrich A8717) at 1:4000 dilution. Arrows indicate the position of the band intensified after HSV1 infection. The intensity of full-length APP bands was appreciably greater than those in the non-transfected cells. In addition the intensity of several smaller bands was also greater than the intensity of the same bands in the mock-transfected (mTf) or normal SHSY5Y (cont), including a band at the same position as the band intensified on HSV1 infection of normal SHSY5Y cells (marked by the arrows), suggested this band is genuinely derived from APP.

On the assumption that a corresponding N-terminal fragment of approximately similar size to that of the 55 kDa C-terminal fragment would be produced as a result of this abnormal processing (the size of APP being 110 kDa to 135 kDa), we probed our blots with the Sigma N-terminal antibody (Fig 3A). However we found no clear evidence for an increased amount of an N-terminal fragment of around this size after infection. Possibly, such a fragment would be either degraded or released into the cell culture medium, thus precluding its detection here.

To confirm further that the C-terminal fragment is derived from APP (and not for example from an APLP, i.e. of a type of protein related to APP, though with a less clear involvement in AD) we obtained human SYSY5Y cells that had been transfected with a human APP gene (APP695), and which over-express APP. We stained blots of proteins from non-transfected, APP transfected, and mock-transfected (i.e. with vector alone) cells with the C-terminal antibody, and found the 55 kDa band in all three cases, though its intensity was greater in the APP transfected cells alone (see Fig. 3B). This indicates that the 55 kDa fragment is indeed an APP product.

## Conclusion

Infection of neuronal cells by HSV1 is known to cause rapid shutoff of protein synthesis prior to viral replication [19]. This is mediated by the virion host shut-off (vhs) protein, which destabilises cellular mRNAs causing them to be degraded. Consequently, and unsurprisingly, transcription of the gene(s) for APP would cease, as would its synthesis and that of other proteins. Our finding that levels of full length APP decline rapidly in infected cells and in cells treated with cycloheximide, suggest that in both cases turnover of full length APP is rapid.

The surprising increase in amount of the 55 kDa C-terminal APP fragment in infected cells shows that processing of APP as well as its synthesis is affected by HSV1, that this occurs within 6 h of infection, and that the level increases with time after infection up to at least 9 h. The size of this fragment and its staining with C-terminal antibody indicate that it includes the region of APP which contains Aβ.

The possibility that the 55 kDa band derives from an APLP or merely reflects a non-specific reaction of the APP antibody with a viral protein or ICP is unlikely. Our viral preparations did not cross-react with this antibody, and at the time when this band begins to increase in intensity (6 h) the majority of ICPs would have already appeared. Furthermore the 55 kDa band is present (at lower levels) in cells not infected with virus. Also, cells transfected with APP (though not mock-transfected cells) have a higher level of this 55 kDa fragment than do untransfected uninfected cells.

The fact that the mock-transfected and cycloheximide-treated cells do not show any intensification of the 55 kDa band suggests that this phenomenon is not due merely to a non-specific stress response, but occurs in response to infection, although the increase in the fragment after HSV2 infection shows the effect is not specific for HSV1 infection alone. Interestingly, infection of human brain microvascular endothelial cells with *Chlamydia pneumonia *(Cpn) may increase the intensity of a similar 55 kDa C-terminal APP band (personal communication, Professor Brian Balin, PA, USA). Thus, increased production of this fragment may be a general response to infection.

Currently HSV1 is the only virus that has been shown to be present in brain of most elderly humans [20] – and may be present as a whole functional genome [21]. Active infection of brain cells with other agents might lead to the same APP effects, but until or unless they are shown to be present, they can not be proposed as possible factors in AD. The situation regarding presence or absence of the bacterium Cpn in brain appears unresolved, although a direct role for Cpn in amyloid systems is supported by recent studies in mice showing amyloid deposition in animals intracerebrally infected with Cpn [22]. Confirmation of Cpn presence would support the possibility that, as with HSV1, the increased level of the 55 kDa fragment that it causes might contribute to AD.

The low levels of Aβ secreted by SHSY5Y cells preclude our examining at present the effects of HSV1 infection on levels of Aβ in cell culture models. Eventual identification of those components of Aβ protein systems which are altered by active viral infection will clarify whether pathogens such as HSV1 can contribute directly to the development of AD neuropathology.

We report here that levels of full length APP rapidly decline in human neuronal type cells acutely infected with either HSV1 or HSV2. Also, the amount of a C-terminal 55 kDa APP fragment which contains the Aβ sequence appears to increase rapidly in infected cells. The fragment level is greater in (non-virally infected) SHSY5Y cells transfected with APP695, suggesting that it is almost certainly derived from APP rather than from APLP. The fragment may increase as part of a host defence mechanism, and/or it might lead to increased generation of Aβ. We are now investigating whether the latter possibility is correct.

## Methods

### Cell culture

Human neuroblastoma (SHSY5Y) cells were maintained in Eagle's minimum essential medium (EMEM) supplemented with 10% (v/v) foetal bovine serum (FBS), 2% (v/v) glutamine and 1% (v/v) penicillin/streptomycin (10% medium), hereafter referred to as growth medium. Cells were incubated at 37°C in a humidified atmosphere of 5% carbon dioxide.

SHSY5Y neuroblastoma cells over-expressing APP_695 _were prepared by double blunt end ligation of the human APP_695 _sequence into the BstXI site of pIREShyg (BD Biosciences Clontech, California, USA). For stable transfections 30 μg of DNA was introduced to cells by electroporation and selection was performed in normal growth medium containing 100 μg ml-1 hygromycin B selection antibiotic (Gibco BRL, Paisley, UK). 'Mock-transfected' cells were stably transfected with the empty pIREShyg vector.

HSV1 (strain SC16) stocks were prepared from a primary stock kindly provided by Prof. Roy Jennings (Department of Virology, University of Sheffield), using Vero cells as host. Herpes simplex virus type 2 stocks were prepared from a clinical isolate kindly provided by Prof Anthony Hart (University of Liverpool) as for HSV1, using HEp2a cells. In both cases, confluent cell monolayers were inoculated at high multiplicities of infection (>10 plaque-forming units (pfu) per cell) with virus suspended in growth medium containing only 1% FBS. Once cytopathic end point was reached (after about two days) virus was harvested from medium and from cells disrupted by low power sonication. Cellular debris was removed by low speed centrifugation (1000 g, 10 min), and virus was isolated by high speed centrifugation of the supernatant (10000 g, 2 h, 4°C) using a Sorvall SS34 rotor. Virus-containing pellets were suspended in PBS, and stored in aliquots at -85°C; their infectivity was assessed by plaque assay of serial dilutions. The virus preparations were checked for bacterial sterility by inoculation into beef heart agar plates and confirming the absence of bacterial growth after incubation for several days at 37°C. For both viruses three sequential passages were prepared, and only passage 3 stocks used here.

SHSY5Y cells were seeded at a concentration of 8 million cells per flask (T175) and incubated overnight. Prior to infection, growth medium was discarded and cells were then washed briefly 10 ml of PBS at 37°C. HSV1 (or in some experiments HSV2) was introduced in 10 ml of growth medium (containing only 0.5% serum), at 3 pfu/cell. For controls, either 0.5% serum containing growth medium alone or the latter containing cycloheximide at 10 μg/ml was used. After 1 hr incubation, the inoculating medium (or control treatment) was removed, and 10 ml of fresh 0.5% serum containing growth medium was added, followed by further incubation for various times.

### Protein extraction and Western blotting

Cells were harvested by removing medium, washing twice with 10 ml PBS, and incubated in 1 mM EDTA (pH 7.4) (in PBS) at room temperature for 10 min. The cell suspension was centrifuged (500 g, 5 min, 4°C), and the cell pellet was resuspended in 400 μl of homogenisation buffer (0.5% Triton X-100 in PBS; 2 mM phenylmethylsulphlfluoride (PMSF); 100 μg/ml of aprotinin and 100 μg/ml leupeptin). Cell lysis was completed by sonication (MSE sonicator, 4°C, 10 μm amplitude, 6 × 10 s).

After measuring the protein concentration of each lysate (BCA protein assay; Pierce), samples were prepared for polyacrylamide gel electrophoresis (PAGE) by mixing 60 μg of protein with 0.25 volume of ×5 Laemmli sample buffer containing 25% β-mercaptoethanol, and boiling for 5 min. Samples were subjected to electrophoresis on 10% SDS-PAGE gels and the proteins transferred to PVDF membranes (Immobilin-P, Millipore) by Western blotting.

Membranes were blocked for 1 h using 8% skimmed-powdered milk in 0.5% Tween in TBS (TBST). The membrane was then washed (this and subsequent washes involved 5 separate 5 min washes in TBS) and then incubated with primary antibody, diluted 1:4000 with TBS (for 1 1/2 h). Primary antibodies used were anti-C-terminal APP (Sigma; A8717), and the anti-N-terminal APP antibody 22C11. After a further wash, membranes were incubated with secondary antibody conjugated with peroxidase (Pierce) which had been diluted 1:1250 with 8% milk in TBS for 1 h. After a final wash the membrane was incubated with Supersignal West Pico Chemiluminescent Substrate Kit (Pierce) for 10 min, and proteins were then visualised by chemiluminescence using a gel documentation system (Syngene).

## Authors' contributions

SJS carried out virology experiments and Western blotting studies, and helped to draft the manuscript. ETP prepared the APP-transfected cells, and participated in the study design. RFI participated in the study design and helped to draft the manuscript. CBD conceived of the study, coordinated it, and drafted the manuscript. All authors read and approved the final manuscript.
